# Pheochromocytoma Secreting Large Quantities of Both Epinephrine and Norepinephrine Presenting with Episodes of Hypotension and Severe Electrolyte Imbalance

**DOI:** 10.7759/cureus.3050

**Published:** 2018-07-25

**Authors:** Amir Shahbaz, Kashif Aziz, Mina Fransawy Alkomos, Usman Nabi, Paria Zarghamravanbakhsh, Issac Sachmechi

**Affiliations:** 1 Internal Medicine, Icahn School of Medicine at Mount Sinai Queens Hospital Center, New York, USA; 2 Research, California Institute of Behavioral Neurosciences & Psychology, Sacramento, USA; 3 Diagnostic Radiology, Hamad General Hospital, Doha, QAT; 4 Endocrinology, Icahn School of Medicine at Mount Sinai Queens Hospital Center, New York, USA

**Keywords:** pheochromocytoma, orthostatic hypotension, hypokalemia, hypocalcemia, epinephrine

## Abstract

Pheochromocytoma is a rare tumor usually arising from the adrenal medulla (strictly speaking, those arising outside the adrenal gland are called paragangliomas). We report a case of pheochromocytoma presenting as orthostatic hypotension and electrolyte imbalance. A 51-year-old woman was admitted because of vomiting and chest pain. She had fluctuating blood pressure (BP) with episodes of orthostatic hypotension. Computed tomography pulmonary angiogram was performed to rule out pulmonary embolism; it showed a clear chest, but an incidental right suprarenal mass. The biochemical analysis supports the diagnosis of pheochromocytoma. Her electrolyte panel revealed persistently low potassium, calcium, and magnesium levels despite aggressive replacement. We speculated that hypotension was mainly due to vasodilatation caused by excess plasma epinephrine and prescribed doxazosin and a nonselective beta-adrenergic blocker which stabilized BP. The right adrenal tumor excised, and postoperatively she remained hemodynamically stable with no hypotensive episode. Laboratory data taken six weeks after surgery show normal 24-hour urine metanephrine and normetanephrine and normal serum magnesium and calcium levels. This case report highlights the variable presentation of pheochromocytoma. We also discuss the probable mechanisms of electrolyte imbalance in our case.

## Introduction

Pheochromocytoma is a catecholamine-secreting neuroendocrine tumor of adrenal or extra-adrenal origin. It usually presents with palpitations, headache, and sweating along with hypertension. The diagnosis is confirmed by increased serum catecholamine levels, and by anatomical localization. The mainstay of definitive therapy is surgical resection [1]. We report a case of pheochromocytoma in a 51-year-old female presented with episodes of hypotension associated with palpitation, sweating, and anxiety. She had electrolyte imbalances including hypokalemia, hypocalcemia, and hypomagnesemia. The abstract of this case had already been presented at a meeting (Abstract: Sachmechi I, Reddy H, Sangsiraprapha W, Lopez R, Reich D, Kim P, Singh G, Pheochromocytoma Secreting Large Quantities of Both Epinephrine and Norepinephrine Presenting with Episodes of Hypotension and Severe Electrolyte Imbalance. The American Association of Clinical Endocrinologists; 2010, https://www.aace.com/files/abstracts-2010.pdf).

## Case presentation

**Figure 1:**
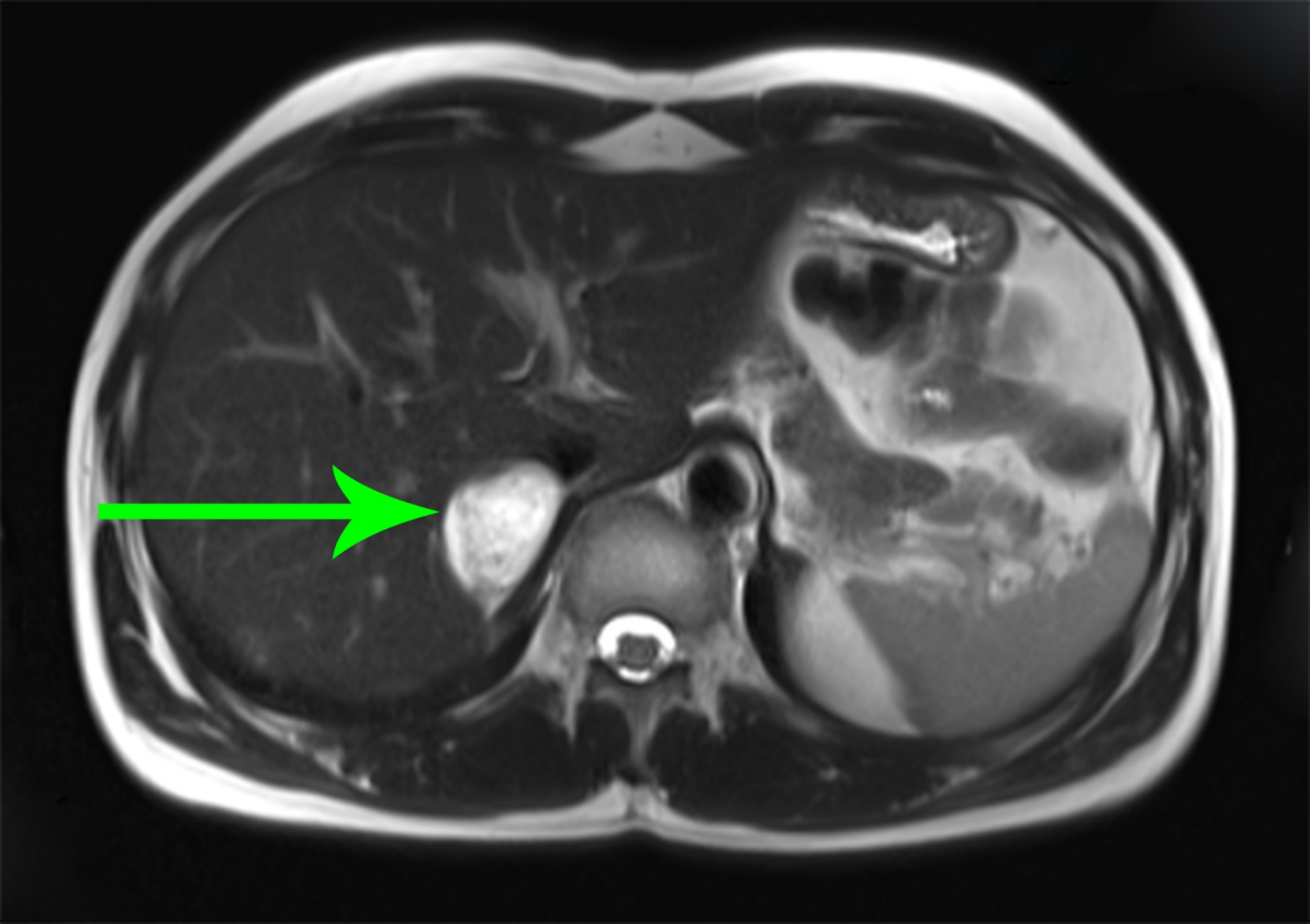
Magnetic resonance imaging (MRI) of the abdomen. The arrow points towards a right suprarenal heterogeneous mass.

A 51-year-old female with a past medical history of type 2 diabetes mellitus presented with complaints of chest pain for three days and vomiting for one day. She had episodes of palpitation, sweating, and weakness for the last three years. These episodes were self-resolving and last for 10-15 minutes. On admission, her blood pressure (BP) was 130/80 mmHg, pulse was 117/min, respiration was 24/min, and oxygen saturation (SpO2) was 100% in room air. While in the hospital she had episodes of orthostatic hypotension with systolic BP ranging from 60 to 130 mmHg and diastolic BP 30-90 mmHg. These episodes were associated with sweating, palpitation, and anxiety and resolved spontaneously. Her pulse remained high ranging between 110 and 120/min. Her electrolyte panel revealed persistently low magnesium, calcium, and potassium levels despite aggressive replacement. She also had persistent hyperglycemia requiring an insulin drip. Computed tomography (CT) angiogram was performed to rule out pulmonary embolism. Incidentally, a large suprarenal hypoattenuating mass was seen pressing on the right kidney. The CT scan of the abdomen with contrast and magnetic resonance imaging (MRI) confirmed it as 11 cm x 11 cm right suprarenal heterogeneous mass as shown in Figure 1.

Further workup along with a normal blood urea nitrogen (BUN), serum creatinine, and parathyroid hormone levels is listed in Table 1:

**Table 1:** Laboratory investigations.

Test	Patient’s value	Normal range
Plasma metanephrine fractioned	3335 pg/mL	<5 pg/mL
Plasma normetanephrine	9355 pg/mL	<148 pg/mL
Serum potassium	3.1 meq/L	3.5-5.5 meq/L
Magnesium	0.6 mg/dL	1.7-2.7 mg/dL
Ionized calcium	3.23 mg/dL	4.25-5.25 mg/dL
Serum corrected calcium	6.2 mg/dL	8.5-10.5 mg/dL
Vitamin D 25 OH	12 ng/mL	20-100 ng/mL
24-hour urine metanephrine	340,000 mcg	90-315 mcg/24 h
24-hour urine normetanephrine	47,552 mcg	112-676 mcg/24 h
24-hour urine dopamine	24 mcg	53-480 mcg/ 24 h
24-hour urine calcium	369 mg	<250 mg/24 h
24-hour urine magnesium	1460 mg	100-150 mg/24 h

**Figure 2:**
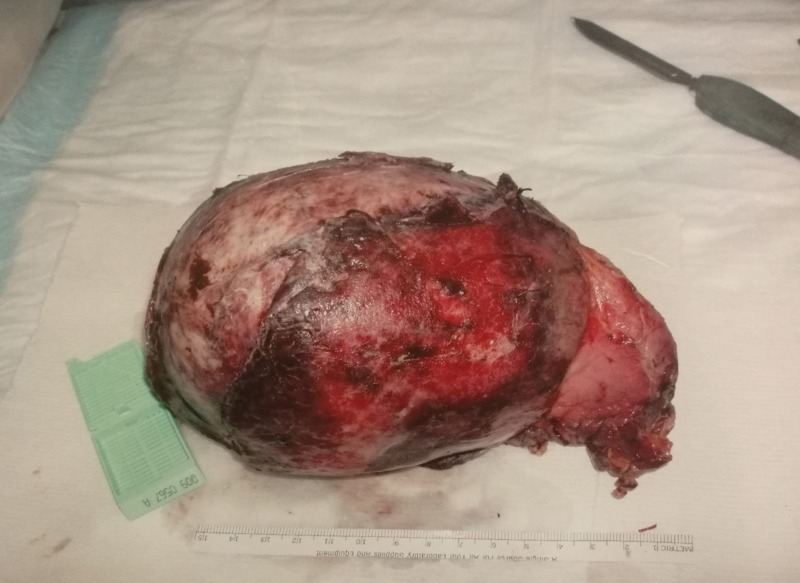
Gross adrenal pheochromocytoma specimen following surgical excision.

**Figure 3:**
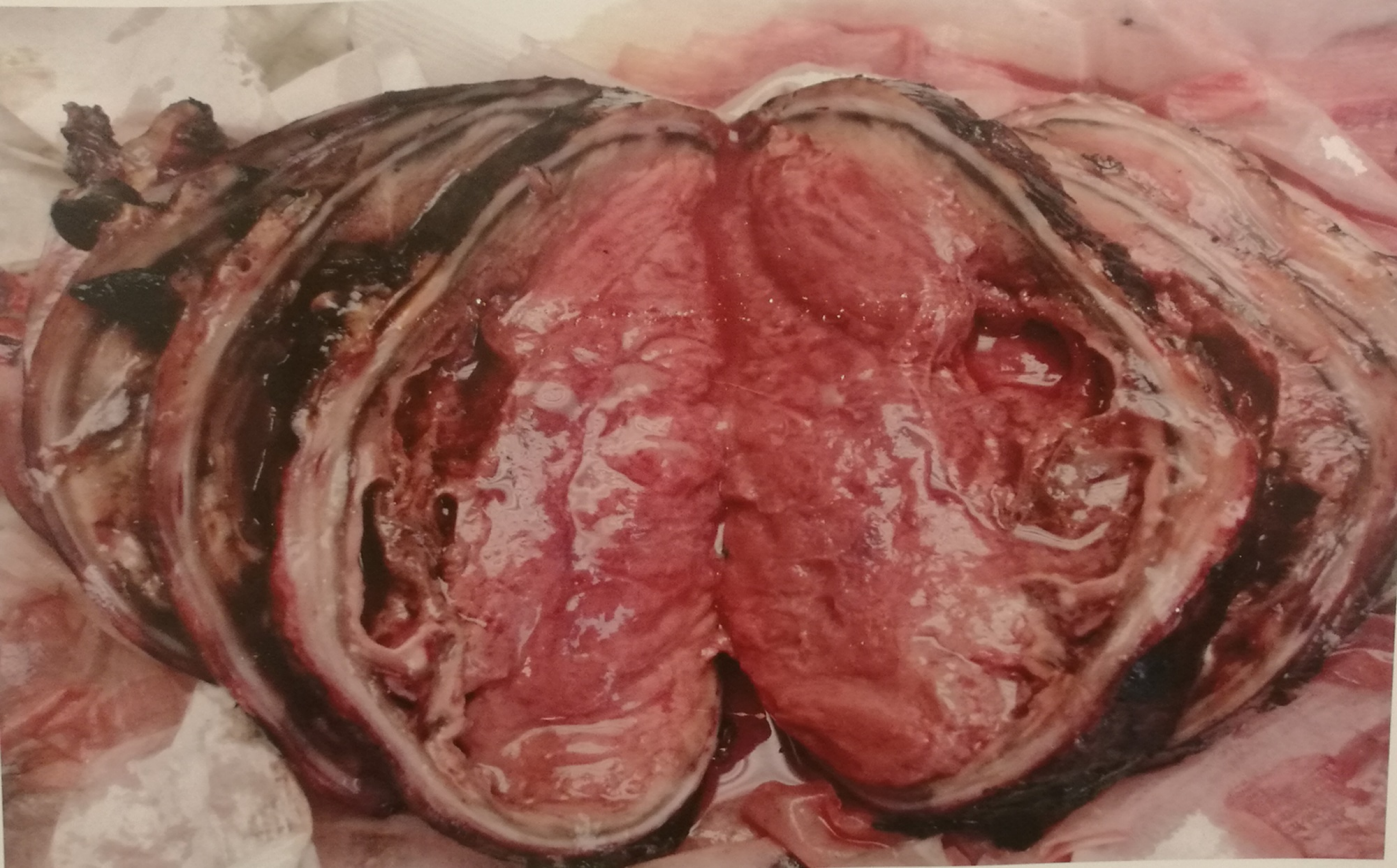
Gross appearance, cut surface. Grayish pink on the cut surface, highly vascularized tumor with areas of necrosis and hemorrhage.

**Figure 4:**
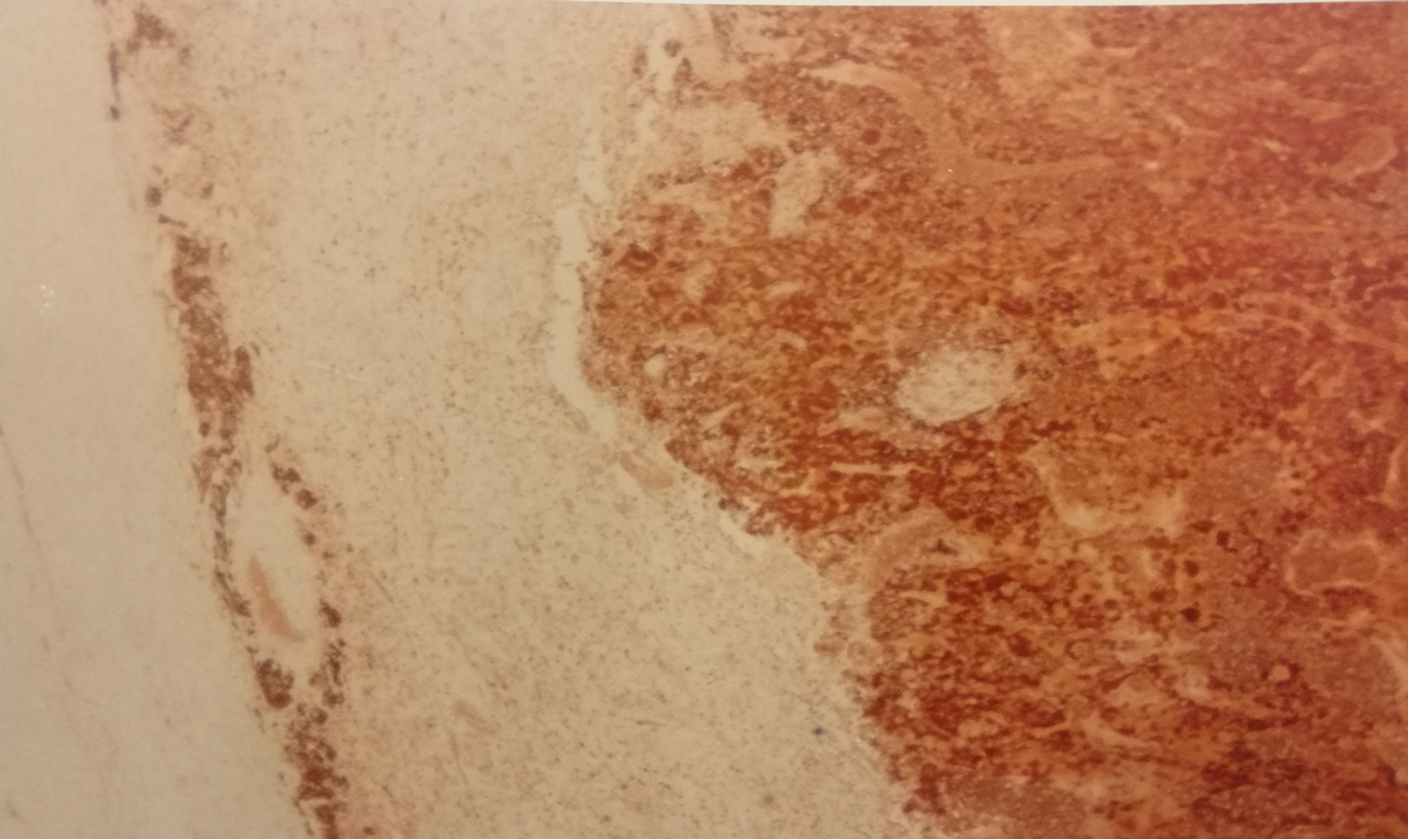
Histology of pheochromocytoma. Histopathological evaluation revealed tumor cells and areas of necrosis.

We made a diagnosis of pheochromocytoma and scheduled her for surgery. Preoperative management consisted of doxazosin 1 mg once daily, propranolol, and IV fluids. Orthostatic hypotension was abated. Three weeks later right adrenalectomy was performed. The pathology revealed a large tumor and a diagnosis of pheochromocytoma. The gross appearance and histology are shown in Figures 2-4.

Postoperatively, she remained hemodynamically stable with no hypotensive episode. Her electrolytes and blood glucose remained normal. Laboratory data taken six weeks after surgery show normal 24-hour urine metanephrine and normetanephrine and normal serum potassium, magnesium, and calcium levels.

## Discussion

Pheochromocytomas are rare, catecholamine-secreting, neuroendocrine tumors usually arising from chromaffin cells of the adrenal medulla. The classic symptoms of pheochromocytoma are headaches, palpitations, anxiety, diaphoresis, and paroxysmal or persistent hypertension [1]. The diagnosis requires biochemical evidence of inappropriate catecholamine production. Measurement of urinary catecholamine levels has traditionally been the most widely used test. Measurements of fractionated metanephrines (i.e., normetanephrine and metanephrine measured separately) in urine and plasma provide superior diagnostic sensitivity to measurements of the parent catecholamines. We prefer CT or MRI for initial localization of a tumor. The test of choice is ^123^I-labeled meta-iodobenzylguanide (MIBG) scintigraphy [2].

In patients with predominantly epinephrine-secreting tumors, symptoms of orthostatic hypotension such as lightheadedness, pre-syncope, and syncope may be more prevalent [1]. The possible mechanisms for orthostatic hypotension due to excess of catecholamine are hypovolemia, intermittent secretion of catecholamines, ratio of epinephrine to norepinephrine in the secretion and impairment of peripheral response to catecholamines [3]. In pheochromocytoma, decreased blood volume caused by consistent vasoconstriction and diminished sympathetic reflex are contributing factors for postural hypotension [4]. Intermittent secretion of catecholamine can cause hypotension because of the increased circulating levels of catecholamine-induced desensitization of the blood vessel to catecholamine and thus abrupt withdrawal of norepinephrine may suddenly decrease the tone of blood vessels [5]. The ratio of epinephrine to norepinephrine plays a vital role in maintaining the BP. Pheochromocytoma secretes several catecholamines. The high plasma epinephrine/norepinephrine ratio indicates that the epinephrine production is augmented compared with norepinephrine. Hypotension is explained by the down-regulation of vascular alpha one adrenergic receptors from exposure to a significant amount of epinephrine resulting in the decrease of the peripheral vascular resistance [6-7]. Vomiting may lead to hypokalemia, but hypokalemia recurred after repletion of potassium and cessation of vomiting. Increased epinephrine concentrations in the plasma secreted by pheochromocytoma induce hypokalemia through stimulation of beta 2 receptors causing activation of sodium potassium-ATPase in skeletal muscles and subsequent intracellular shift of potassium [8-9]. There was an association of hypokalemia and potassium depletion with decreased magnesium absorption within the loop and distal tubule that may lead to increased magnesium excretion [10]. Increased urinary loss of calcium due to hypomagnesemia and hypokalemia explains profound hypocalcemia requiring continuous intravenous calcium repletion. Pheochromocytomas are known to secrete adrenomedullin. Its physiological actions include increased calcium sequestration within the bones, leading to hypocalcemia. Adrenomedullin is similar to the calcitonin family and stimulates osteoblast activity and bone mineralization. Also, it has potent vasodilatory effects in several vascular systems, which may contribute to the hypotensive episodes [11].  

Pheochromocytoma should receive appropriate preoperative medical management to block the effects of released catecholamines. For preoperative blockade, there is no specific recommendation on the preferred drugs; alpha-adrenoceptor antagonists, calcium-channel blockers, or angiotensin-receptor blockers have all been recommended and appear useful. We recommend beta-adrenoceptor or calcium-channel blockers for tachyarrhythmias. Beta-adrenoceptor blockers have to be used only after adequate pretreatment with alpha-adrenoceptor antagonists. Adequate volume expansion before and after surgery is essential. The use of laparoscopy as the surgical method of choice for most abdominal pheochromocytomas is recommended for primary or multiple smaller tumors [2]. There should be a regular postoperative follow-up visit. Follow-up in unilateral adrenal disease with no genetic predisposition is once a year, as a clinical and biochemical follow-up for a total of 10 years. The first control takes about six weeks after the operation. If there is a genetic mutation, the tumor is large, or the patient is very young at initial diagnosis, lifelong follow up is recommended [12].

## Conclusions

Although most pheochromocytomas present with episodes of hypertension, rarely they can present with hypotensive episodes. Alpha-receptors antagonists have to be always used before beta-blockers for BP control and correction of hypovolemia before and after surgery is essential. The clinician should be aware of the possibility that hyperglycemia, hypokalemia, hypomagnesemia, and hypocalcemia can be part of the presentation of pheochromocytoma in the absence of hypertension.
